# Always Connected, Not Always Well: The Moderating Role of Sleep Quality in the Relationship Between Nomophobia and Mental Health Among University Students in Saudi Arabia

**DOI:** 10.3390/ejihpe16090140

**Published:** 2026-09-15

**Authors:** Ibrahim A. Elshaer, Alaa M. S. Azazz, Chokri Kooli

**Affiliations:** 1 Management Department, College of Business Administration, King Faisal University, Al-Ahsa 31982, Saudi Arabia; 2 Department of Social Studies, Arts College, King Faisal University, Al-Ahsa 31982, Saudi Arabia; aazazz@kfu.edu.sa; 3 Department of Management, Faculty of Social Sciences and Humanities, Royal Military College of Canada, Kingston, ON K7K 7B4, Canada; ckooli@uottawa.ca; 4 Graduate School of Public and International Affairs, Faculty of Social Sciences, University of Ottawa, Ottawa, ON K1N 6N5, Canada

**Keywords:** nomophobia, sleep quality, depression, anxiety, stress, mental health, university students, PLS-SEM

## Abstract

As smartphones become progressively embedded in students’ social, academic, and personal lives, concerns have been raised about the emotional consequences of nomophobia (no-mobile-phone phobia). Although previous research has shown an overall correlation between nomophobia and increased mental health symptoms, little is known about whether its main dimensions exerted differential associations with different mental health symptom dimensions or whether sleep quality can influence these relationships. Addressing these gaps, this research tested the associations between four dimensions of nomophobia—Not Being Able to Communicate (NBAC), Losing Connectedness (LC), Not Being Able to Access Information (NBAI), and Giving Up Convenience (GUC)—and three mental health symptoms: depression (Dprtn), anxiety (Enzt), and stress (Strs), while testing the moderating role of sleep quality (SQ) among university students. Drawing upon Conservation of Resources (COR) Theory and Self-Determination Theory (SDT), a quantitative cross-sectional survey was conducted among 980 university students. The proposed research model was analyzed using Partial Least Squares Structural Equation Modeling (PLS-SEM). The results demonstrated that the psychological consequences of nomophobia are dimension-specific rather than homogeneous. LC emerged as the most consistent predictor, showing significant positive associations with depression, anxiety, and stress. In contrast, NBAC was not significantly associated with any of the three mental health outcomes. NBAI showed mixed effects, with a significant positive association with depression, a significant negative association with anxiety, and no significant association with stress. GUC was significantly and positively associated with depression, anxiety, and stress. Furthermore, sleep quality functioned as a selective moderator, with significant interaction effects for SQ × NBAC on depression and anxiety, SQ × NBAI on depression, and SQ × GUC on depression and stress. No significant moderation effects were observed for the remaining hypothesized interactions. These findings highlight the dimension-specific nature of nomophobia and indicate that the role of sleep quality varies across the specific nomophobia dimension and the mental health outcome considered.

## 1. Introduction

Smartphones have increasingly become an indispensable means of communication, information access, entertainment, social interaction, and academic practice (Bijlsma et al., 2019; Han & Yi, 2019). University undergraduates are among the most intensive users of smartphones, and their daily habits are increasingly shaped by constant digital connectivity (Montag et al., 2021). While smartphones can provide substantial social and educational benefits, excessive reliance on them has raised several challenges for digital mental health and well-being (Demirci et al., 2015). One of the most pronounced manifestations of such reliance is nomophobia, described as the fear of being unable to use or access smartphones (Yildirim & Correia, 2015). The growing use of smartphones has been associated with an increase in problematic smartphone use and technology-related problems, particularly among adults and higher education students (Bijlsma et al., 2019). Nomophobia (no-mobile-phone phobia) (Bragazzi & Del Puente, 2014) is not just a behavioural issue; it reflects psychological discomfort associated with perceived digital discontinuation and may include worries about losing communication and convenience (Bhattacharya et al., 2019; Yildirim & Correia, 2015). Emerging evidence shows that higher levels of nomophobia are correlated with symptoms of depression, anxiety, stress, and decreased well-being (Adawi et al., 2018; Alhusseini et al., 2025; Daraj et al., 2023; Jahrami et al., 2021; León-Mejía et al., 2021; Rawas et al., 2021). These results suggested that nomophobia may constitute a critical emotional risk factor among university students (Tuco et al., 2023).

University students’ mental health has become a significant public health concern worldwide. University students repeatedly face academic challenges, career ambiguity, and social adjustment pressures, all of which might foster vulnerability to emotional distress (Auerbach et al., 2018; Elshaer et al., 2026b). Recent evidence showed that symptoms of mental health, such as depression, anxiety, and stress, are common among university students and are exacerbated in the setting of intensive digital involvement (Gnardellis et al., 2023; Zhuang & Jenatabadi, 2023). Understanding digital-related predictors of mental health among university students is thus a critical research priority. One element that might play a significant role in these relationships is sleep quality (Elkin et al., 2025). Sleep is a basic biological and psychological process that regulates emotional adaptation, cognitive functioning, and psychological flexibility (Buysse, 2014). Poor sleep quality has been consistently associated with depression, anxiety, stress, and decreased well-being among higher education students (Alfonsi et al., 2020; Lund et al., 2010). Excessive smartphone use and nighttime digital encounters might disrupt sleep by inducing intellectual arousal, psychological stimulation, delayed sleep onset, and prolonged exposure to screen light (Alfonsi et al., 2020). Subsequently, sleep quality might serve as a principal mechanism through which digital engagement can be associated with mental health. Despite growing interest in nomophobia, several limitations persist in the literature. First, several research has primarily investigated only the direct link from nomophobia to mental health outcomes (Adawi et al., 2018; Alhusseini et al., 2025; Bano et al., 2021; Gnardellis et al., 2023; Han & Yi, 2019; Kumar et al., 2025; León-Mejía et al., 2021; Li et al., 2020; Rawas et al., 2021), without any moderations. Second, sleep quality has frequently been examined as an outcome of smartphone overuse rather than as a moderating factor that might alter the link between nomophobia and mental health symptoms (Alfonsi et al., 2020; Demirci et al., 2015; Sadeghi et al., 2025). Third, few studies have tested these factors simultaneously within a single integrated model targeting university students, particularly in non-Western contexts. As a result, it remains unclear whether good sleep quality can buffer the adverse psychological effects of nomophobia or whether poor sleep quality exacerbates them. Filling this gap is theoretically essential. Based on the Conservation of Resources (COR) theory, sleep quality might be hypothesized as a personal asset that helps people maintain psychological and cognitive performance (Hobfoll, 1989). Nomophobia might exhaust emotional resources by creating continued anxiety about digital detachment, whereas adequate sleep might replenish these assets and decrease vulnerability to distress. Investigating sleep quality as a moderator can consequently offer a better understanding of the factors under which nomophobia becomes emotionally harmful. The current study is motivated by three main considerations. First, the frequency of smartphone reliance among university students is growing globally (Montag et al., 2021). Second, mental health symptoms among university students represent a persistent public health and educational challenge (Auerbach et al., 2018). Third, sleep disturbances are widespread among student populations and may be mitigated through protective interventions (Lund et al., 2010). Exploring the interrelationships between nomophobia dimensions (not being able to communicate, losing connectedness, not being able to access information, and giving up convenience), sleep quality, and mental health symptom dimensions (depression, anxiety, and stress) might therefore create evidence that is both theoretically expressive and practically relevant. Accordingly, the main objective of this study is to investigate the association between nomophobia and mental health among university students and to test whether sleep quality moderates this relationship.

The remainder of this paper is organized as follows. Section 2 reviews the literature and develops the research hypotheses. Section 3 describes the research methods, including sample size and selection, measures of the study factors, and the analytical methods employed. Section 4 presents the empirical findings, while Section 5 interprets the results in relation to current literature and theoretical Lenses. Finally, Section 6 concludes with a discussion of limitations and future research directions.

## 2. Literature Review and Hypotheses Development

Nomophobia has been identified as one of the most prominent phenomena of smartphone reliance among adults, particularly university students, whose social, academic, and daily routines increasingly depend on constant smartphone use (Bragazzi & Del Puente, 2014). Formerly coined by Yildirim and Correia (2015), nomophobia describes the “fear or anxiety experienced when individuals are unable to use or access their smartphones”. Based on multiple evidence-based studies using qualitative, quantitative, and mixed methodologies, the authors (Yildirim & Correia, 2015) stated four main dimensions of nomophobia: “Not Being Able to Communicate” (NBAC), “Losing Connectedness”, “Not Being Able to Access Information”, and “Giving Up Convenience”. NBAC represented the psychological distress correlated with losing the capability to sustain instant communication with peers, family, or others. As smartphones have become an essential method for interpersonal communication, social interaction, and psychological reassurance, communication deficiency might trigger negative psychological reactions, mostly among university students who depend heavily on digital interaction in their social and academic lives (Adawi et al., 2018; Rodríguez-García et al., 2020; Yildirim & Correia, 2015).

The current study built on an integrated theoretical approach combining Conservation of Resources (COR) Theory, Self-Determination Theory (SDT), Compensatory Internet Use Theory (CIUT), and the Hyperarousal Model to understand the relationships between nomophobia, mental health symptoms, and sleep quality. COR Theory (Hobfoll, 1989) offered the overarching foundation by proposing that people experience psychological strain when valued personal, social, and psychological resources are threatened, depleted, or perceived as difficult to sustain. In the context of nomophobia, issues about being unable to communicate, losing connectedness, being unable to access information, or giving up the convenience offered by smartphone connectivity might represent perceived threats to valued resources and might therefore be correlated with greater depressive, anxiety, and stress symptoms. SDT, as introduced by Ryan and Deci (2000) can complement this point of view by highlighting the importance of essential psychological needs, specifically relatedness, autonomy, and competence, which might increase the perceived importance of continuous smartphone-mediated connectivity. CIUT can further suggest that people might depend on internet- or smartphone-mediated activities to compensate for unmet psychological needs or negative emotional states, potentially reinforcing reliance on continuous connectivity (Kardefelt-Winther, 2014). Finally, the Hyperarousal Model offers a complementary explanation for the role of sleep quality, suggesting that heightened cognitive and emotional arousal is associated with difficulties in obtaining restorative sleep and greater psychological strain (Bonnet & Arand, 2010; Riemann et al., 2010). The relationship between NBAC and mental health symptoms (MHS) can be understood through the lens of Conservation of Resources (COR) Theory (Hobfoll, 1989), which suggests that emotional distress results when people perceive a risk of losing a valuable personal asset. In the digital age, smartphones have evolved beyond mere communication tools into a must-have social resource that facilitates interpersonal communication. Subsequently, perceived failure to communicate might lead to a loss of these assets, causing psychological strain and emotional vulnerability (Aftab & Khyzer, 2023; Bijlsma et al., 2019). Likewise, the Compensatory Internet Use Theory argued that people progressively relied on digital communication tools to regulate adverse emotions and sustain psychological well-being, making communication disturbance principally distressing for psychologically vulnerable people (Kardefelt-Winther, 2014). Previous empirical evidence progressively supported the correlation between nomophobia and MHS. Numerous studies have confirmed that people with higher levels of nomophobia showed significantly higher levels of MHS, such as depression, anxiety, and stress (Kumar et al., 2025; León-Mejía et al., 2021). Systematic reviews have also concluded that nomophobia is constantly associated with a wide range of negative psychological consequences, including psychological distress, loneliness, and reduced levels of quality of life (Daraj et al., 2023; Elhai et al., 2017; Gardani et al., 2022). More precisely, recent studies among university students have reported that fear of being detached from smartphone devices is correlated with higher symptom levels of depression, anxiety, and stress, indicating that excessive emotional dependence on smartphones might undermine psychological regulation and emotional resilience (Kumar et al., 2025; Tolan & Karahan, 2022; Tuco et al., 2023). Despite this evidence, some limitations remain in current literature. First, most previous research has conceptualized nomophobia as a unidimensional global construct, depending on the average NMP-Q score rather than investigating the distinctive emotional impacts of its factors (Valenti et al., 2024). Subsequently, little is known about whether the NBAC dimension of Nomophobia can exert a unique relationship with specific MHS (depression, anxiety and stress) beyond the overall average of the nomophobia construct. Testing the specific contribution of the NBAC dimension then can offer a more improved understanding of how distinctive factors of nomophobia contribute to MHS among university students. Accordingly, the following hypotheses are introduced:

**H1.**  *NBAC (as a dimension of nomophobia) can be positively and significantly associated with depression symptoms.*

**H2.**  *NBAC (as a dimension of nomophobia) can be positively and significantly associated with anxiety symptoms.*

**H3.**  *NBAC (as a dimension of nomophobia) can be positively and significantly associated with stress symptoms.*

Among the four dimensions of nomophobia, “Losing Connectedness” (LC) conceptualises people’s fear of being disconnected from their online identity, social connections, and digital society when they are unable to access smartphones (Yildirim & Correia, 2015). Unlike the other nomophobia dimensions, which emphasise the failure to make calls or exchange instant messages, the LC dimension highlights the broader emotional concern of losing constant connection to one’s digital social setting (León-Mejía et al., 2021). As smartphones increasingly serve as gateways to social interaction platforms, instant messaging applications, and online communities, detachment may create feelings of exclusion, isolation, and ambiguity that negatively affect emotional well-being (Nikolic et al., 2023). The association between LC and MHS can be understood through the Lens of the COR Theory (Hobfoll, 1989), which suggests that people experience emotional distress when they lose valuable resources. In digitally connected communities, online connections and constant social interactions provide important emotional resources, offering psychological support, connectedness, and social validation (Bijlsma et al., 2019). Accordingly, perceived detachment from these online networks might represent a significant asset loss, causing a heightened psychological distress (Li et al., 2020). This approach is further reinforced by Self-Determination Theory (SDT), which suggests that the main emotional need for relatedness is essential for sustaining emotional well-being (Ryan & Deci, 2000). Smartphones have been identified as one of the main tools through which higher education students meet this need by sustaining continuous interaction with friends, family members, and the wider social world (Amez & Baert, 2020). Consequently, the fear of losing connection might threaten people’s feelings of belonging and contribute to adverse psychological consequences (Demirci et al., 2015).

Previous empirical research has consistently shown that nomophobia (as a unidimensional construct) is positively correlated with MH symptoms such as depression, anxiety, and stress among university students (Gnardellis et al., 2023; Tolan & Karahan, 2022). These studies showed that students displaying higher levels of fear from smartphone disconnection reported significantly poorer MHS across multiple psychological settings. Nevertheless, relatively little research has explored whether single dimensions of nomophobia might contribute differently to these outcomes. Recent research has started to address this issue. A cross-sectional study conducted among Saudi Arabia university students explored the four dimensions of the NMP-Q separately. They argued that LC, together with the other dimensions, was significantly associated with depression, anxiety, stress, and overall emotional distress operationalized using the DASS-21 scale (Bano et al., 2021). Employing the four different dimensions of nomophobia as reported in the NMP-Q, the authors stated that the LC dimension exhibited one of the strongest positive associations with depression symptoms and was also positively correlated with stress and anxiety (Bano et al., 2021). The authors stated that excessive reliance on maintaining constant digital social connections might increase psychological vulnerability, particularly among adults whose personal and academic lives are directly integrated with smartphone-mediated connections. Evidence from previous systematic reviews supported these findings. León-Mejía et al. (2021) argued that nomophobia is consistently correlated with several indicators of poor emotional health such as depression, anxiety, and stress. Although the review synthesized results based mainly on overall nomophobia average score rather than single dimensions, the authors stated that fear of social disconnection signifies one of the main mechanisms through which nomophobia is adversely associated with mental health. From a theoretical lens, university students who are subjected to higher levels of fears of losing their digital social interaction are more likely to perceive higher levels of social isolation and condensed psychological support when smartphone access is unobtainable (Rodríguez-García et al., 2020). Accordingly, the following hypotheses can be presented:

**H4.**  *LC (as a dimension of nomophobia) can be positively and significantly associated with depression symptoms.*

**H5.**  *LC (as a dimension of nomophobia) can be positively and significantly associated with anxiety symptoms.*

**H6.**  *LC (as a dimension of nomophobia) can be positively and significantly associated with stress symptoms.*

Not Being Able to Access Information (NBAI)**,** as an aspect of nomophobia, describes the discomfort and fear people experience when they are unable to obtain information through their smartphones (Yildirim & Correia, 2015). This dimension highlights concerns about losing instant access to online news, information, and other digital platforms that have become fundamental to everyday activities (Bragazzi & Del Puente, 2014). Unlike other communication-related aspects of nomophobia, NBAI primarily emphasises reliance on smartphones as a means of information processing, highlighting people’s increasing dependence on mobile devices to reduce ambiguity, solve daily problems, and support decision-making (León-Mejía et al., 2021). The emotional implications of NBAI can be interpreted through the lens of COR Theory (Hobfoll, 1989), which argues that stress arises when people experience actual resource loss. Therefore, the inability to retrieve information on mobile devices might be perceived as a loss of informational assets, thereby increasing uncertainty and psychological discomfort. The initial development of the NMP-Q acknowledged NBAI as one of the four main dimensions of nomophobia, noting that people repeatedly reported nervousness and discomfort when they could not immediately access information on their mobile devices (Adawi et al., 2018).

Several pieces of evidence supported the broader association between nomophobia and MHS. Çakmak Tolan and Karahan (Tolan & Karahan, 2022) found that higher levels of nomophobia can significantly predict depression, anxiety, and stress among university students, signifying that extreme psychological reliance on smartphones forms a critical risk factor for psychological distress. Additionally, a meta-analysis review by Daraj et al. (2023) and Sheikh et al. (2024) concluded that a moderate to severe nomophobia is steadily correlated with depression, anxiety, stress, loneliness, and decreased well-being across university students, supporting the wider emotional implications of smartphone-related overuse. From a theoretical perspective, students who rely heavily on smartphones for information access might experience greater uncertainty, weaken personal control, and encounter cognitive challenges when this resource is unavailable (Jahrami et al., 2021). These reactions might successively contribute to depression symptoms through a sense of vulnerability, to anxiety through worsened uncertainty and worry, and to stress through perceived failure to control academic and daily demands and duties (Nikolic et al., 2023). Accordingly, the following hypotheses can be proposed:

**H7.**  *NBAI (as a dimension of nomophobia) can be positively and significantly associated with depression symptoms.*

**H8.**  *NBAI (as a dimension of nomophobia) can be positively and significantly associated with anxiety symptoms.*

**H9.**  *NBAI (as a dimension of nomophobia) can be positively and significantly associated with stress symptoms.*

Giving Up Convenience (GUC), as a dimension of nomophobia echoes people’s discomfort arising from the perceived loss of the practical efficiency and benefits that smartphones offer in daily life (Yildirim & Correia, 2015). GUC describes smartphones as multifunctional devices that can facilitate routine and daily practices such as online banking, meeting scheduling, shopping, and task management (León-Mejía et al., 2021). Yildirim and Correia (2015) stated that applicants described smartphones as offering a feeling of “peace of mind” because they can simplify everyday activities and minimise uncertainty. The relationship between GUC and MHS can be interpreted through COR Theory (Hobfoll, 1989), which suggests that people experience emotional strain when valuable assets are threatened. In the digital age, smartphone devices have become indispensable individual resources that minimize cognitive effort, enhance efficiency, and facilitate daily decision-making. Accordingly, losing the benefits offered by smartphone devices might be perceived as a loss of a main resource that maximizes emotional strain and minimizes people’s perceived capability to cope with everyday challenges (Elhai et al., 2017). Previous empirical evidence has gradually supported the broader association between nomophobia (as a unidimensional construct) and negative MHS (Alhusseini et al., 2025; Rawas et al., 2021). Numerous research has showed that higher levels of nomophobia are correlated with higher levels of depression, anxiety, and stress symptoms among university students (Gnardellis et al., 2023; Tolan & Karahan, 2022). Qualitative evidence further supported these conclusions. During the initial development of the NMP-Q, applicants frequently depicted smartphones as necessary tools that can simplify everyday life and minimize uncertainty (Rodríguez-García et al., 2020). Several contributors reported experiencing pressing stress and anxiety when their smartphone battery became drained because they identified themselves as losing access to fundamental daily activities (Bhattacharya et al., 2019).

From a theoretical perspective, university students who depend heavily on smartphones to organise their social, academic, and personal activities might experience heightened emotional distress when these conveniences become inaccessible (Han & Yi, 2019). The unexpected loss of smartphone support might minimize perceived control over daily duties, increase ambiguity, and increase cognitive burden (Lin et al., 2021). Such experiences might foster depressive symptoms through a sense of helplessness and decreased self-efficacy, anxiety symptoms through anticipatory issues concerning task completion and indecision, and stress symptoms through amplified perceived challenges and weakened coping resources (Basu et al., 2025). Accordingly, the following hypotheses can be proposed:

**H10.**  *GUC (as a dimension of nomophobia) can be positively and significantly associated with depression symptoms (as a dimension of mental health symptoms).*

**H11.**  *GUC (as a dimension of nomophobia) can be positively and significantly associated with anxiety symptoms (as a dimension of mental health symptoms).*

**H12.**  *GUC (as a dimension of nomophobia) can be positively and significantly associated with stress symptoms (as a dimension of mental health symptoms).*

Sleep quality has increasingly been recognised as one of the most important contributors to emotional well-being among higher education students (Nelson et al., 2022). The shift to university life is frequently accompanied by increased academic demands, lifestyle challenges, and excessive technology use, all of which can contribute to sleep instability (Hershner & Chervin, 2014; Lund et al., 2010). Because smartphones have become deeply rooted in students’ academic life, social interactions, and recreational practices, concerns about smartphone departure might be negatively correlated with sleep quality, which can adversely affect psychological well-being (Demirci et al., 2015).

From a theoretical point of view, the moderating role of sleep quality can be understood through COR Theory (Hobfoll, 1989). COR theory posits that people strive to preserve valuable resources, and stress can occur when ownership of these resources is depleted (Halbesleben et al., 2014). Sleep quality was among the most important resources because it replenishes cognitive functioning, emotional adaptation, and psychological flexibility (Nelson et al., 2022). People with high levels of nomophobia continually remain psychologically preoccupied with smartphone accessibility, receive notifications, and constant connectivity, thus worsening their competence to recover restorative sleep (Sadeghi et al., 2025). Over time, sleep deficiency minimizes people’s ability to cope with everyday challenges, maximizing their vulnerability to depression, stress, and anxiety (Demirci et al., 2015). Subsequently, sleep quality emerged as a possible moderator linking nomophobia with MHS.

Most current studies have mainly investigated the direct correlation between nomophobia and MHS symptoms such as depression, anxiety, and stress, while comparatively few studies have tested the boundary conditions under which these associations become stronger or weaker. Among the possible contextual elements, sleep quality has received limited attention as a moderating factor, despite extensive evidence of its central role in emotional functioning and psychological regulation. Furthermore, previous research has generally conceptualised nomophobia as a unidimensional factor (Valenti et al., 2024), overlooking the possibility that its four distinct theoretical dimensions might interact differently with sleep quality in predicting MHS. This distinction is theoretically significant because each dimension shows a unique pattern of smartphone reliance that may differ in its vulnerability to the defensive or worsening effects on sleep quality. For example, university students characterized by NBAC might experience continuing concerns about skipping messages or calls, causing heightened levels of cognitive arousal that become more emotionally harmful when sleep quality is poor (Adawi et al., 2018). People who experience LC might engage in constant social media use and pursue continuous online social reassurance, actions that might contribute to psychological distress, regardless of sleep quality (Yildirim & Correia, 2015). Similarly, university students with high levels of NBAI might suffer from anxiety arising from limited access to online information, even though adequate sleep might enhance psychological regulation and minimize the emotional effect of such concerns (Tadros et al., 2025). Likewise, people characterised by GUC might depend heavily on smartphones for managing daily challenges, making them particularly vulnerable to emotional distress when poor sleep compromises cognitive processes, self-control, and coping ability (Bhattacharya et al., 2019). Drawing on COR Theory, which argues that individual resources buffer people against emotional strain (Hobfoll, 1989), and the “Hyperarousal Model of Insomnia”, which highlighted the role of sleep disturbances in damaging emotional regulation (Bonnet & Arand, 2010; Riemann et al., 2010), this research proposed that the mental health outcomes of nomophobia are contingent upon people’s sleep quality. Specifically, poor sleep quality is expected to amplify the negative emotional effects of smartphone reliance, whereas good sleep quality might mitigate these associations by facilitating psychological resilience and adaptive coping. Accordingly, the following moderation hypotheses can be proposed:

**H13a–H13l.**  *Sleep quality can moderate the link from nomophobia’s four dimensions to mental health three dimensions.*

## 3. Methods

### 3.1. Research Design

This research employed a cross-sectional survey, using a structured questionnaire to explore and test the hypothesised associations among the study’s main factors. Such a technique is appropriate, as it can facilitate the collection of the required data from a large number of targeted participants within a limited timeframe and permit the analysis of complex associations using the PLS-SEM technique (Hair et al., 2021) with SmartPLS v4 (GmbH, Bönningstedt, Germany). The selection of PLS-SEM was not based solely on model complexity or sample size. Rather, it was driven by the study’s main objective: explaining variance in multiple endogenous mental-health consequences while simultaneously estimating the dimension-specific associations with four nomophobia dimensions and the conditional effects of sleep quality via multiple interaction terms. PLS-SEM is particularly appropriate for models that emphasise explained variance and predictive relevance, and that include multiple dependent dimensions and moderating variables. Although the hypothesised relationships were derived from a well-established theoretical background, the current study also aims to identify the extent to which the assumed nomophobia dimensions can explain variance in depression, anxiety, and stress, and whether sleep quality can enhance the predictive performance of these associations.

### 3.2. Participants

Data were obtained from undergraduate university students enrolled at five major universities across different regions of Saudi Arabia (SA). The process of collecting the study sample was designed to capture students from various regional contexts. Specifically, 29% of the participants were enrolled at King Faisal University (KFU; Eastern Region), followed by 24% from Umm Al-Qura University (UQU; Western Region), 23% from Imam Mohammad ibn Saud Islamic University (IMAMU; Central Region), 13% from Jazan University (JazanU; Southern Region), and 11% from Northern Border University (NBU; Northern Region).

A total of 1000 forms were circulated. Respondents were subjected to three inclusion conditions: participants had to (1) currently be enrolled at SA University, (2) use a smartphone daily, and (3) provide voluntary informed consent before answering the questionnaire. Answers were removed from the dataset when questionnaires were incomplete, contained substantial missing information, or were submitted by individuals who did not fulfill the student-status requirement at the time of data collection. Before participation, respondents were provided with information about the research’s aim and assured that their responses would remain anonymous and confidential.

The initial screening removed 20 responses due to incomplete responses, excessive missing information, or failure to meet eligibility requirements. Consequently, 980 usable questionnaires were retained for empirical analysis, representing a valid response rate of 98.0% from the distributed forms.

Regarding academic background, students enrolled in social-science disciplines—including business, arts, and law—accounted for 45% of the final sample. The remaining 55% were enrolled in natural and applied science disciplines, including computer science, engineering, agriculture and food sciences, and health-related programs. The gender distribution consisted of 60% male and 40% female respondents. With respect to age, the sample was predominantly composed of younger university students: 90% were between 17 and 21 years old, whereas the remaining 10% were older than 21.

Overall, the sampling design incorporated students from multiple Saudi universities, geographical regions, academic disciplines, genders, and age groups. This diversity provides an appropriate empirical context for examining the relationship between nomophobia and mental health and the moderating role of sleep quality among university students in Saudi Arabia.

### 3.3. Scale and Procedures

Nomophobia was operationalised using 20 items measuring four dimensions, as suggested by Yildirim and Correia (2015), in the Nomophobia Questionnaire (NMP-Q). The four dimensions are entitled Not Being Able to Communicate (NBAC-6 items), Losing Connectedness (LC-5 items), Not Being Able to Access Information (NBAI-4 items), and Giving Up Convenience (GUC-5 items). Sample items of the four dimensions, in the same order, include, “I would feel anxious because I could not instantly communicate with my family-and/or freinds”, “I would be nervous because I would be disconnected from m online identity”, “1 would be annoyed if I could not look up information on my smartphone”, and “Running out of battery in my smartphone would scare me”. Similarly, MHS was operationalized using the depression, anxiety stress scale (DASS-21) with three main factors as suggested by Lovibond (Lovibond, 1995). The Arabic adaptation of the DASS-21 has also been examined in Arabic-speaking populations, with evidence supporting its applicability and psychometric properties across relevant samples (Elshaer et al., 2026a, 2026b; Elshaer & Azazz, 2023, 2025). The answer options of DASS-21 scale are between 0 = “Did not apply to me at all” to 3 = “Applied to me very much or most of the time”. Respondents were asked to show the level to which they have symptoms (stress, anxiety, and depression) of MHS during a specified period (last 7 days). Each dimension (stress, anxiety, and depression) of the DASS-21 scale has 7 variables, sample items include, in the same order, “I was unable to become enthusiastic about anything”, “I felt close to panic”, “I found it difficult to calm down”. Finally, sleep quality (SQ) was measured using six items from Yu et al. (2012) that assessed participants’ sleep experiences over the last 7 days. The six items assessed: (1) “In the past 7 days, my sleep quality was,” with reply options ranging from 5 = “Very Poor” to 1 = “Very Good”; (2) “In the past 7 days, my sleep was refreshing,” ranging from 5 = “Not at all” to 1 = “Very Much”; (3) “In the past 7 days, I had a problem with my sleep”; (4) “In the past 7 days, I had difficulty falling asleep”; (5) “In the past 7 days, my sleep was restless”; and (6) “In the past 7 days, I tried hard to get to sleep,” with Items 3–6 coded from 1 = “Not at all” to 5 = “Very Much.” Because the original response coding results in higher raw scores suggesting poorer sleep or greater sleep disturbance, all six items were reverse-scored before constructing the SQ measure using the transformation (6 − raw score). Accordingly, higher SQ scores in the current study represent better sleep quality. This scoring procedure ensured that the direction of the SQ construct was conceptually consistent across all six indicators and with the interpretation of the moderation effects.

The questionnaire underwent a thorough translation process to guarantee linguistic clarity and conceptual equivalence. Initially, the English-language scale was translated into Arabic by a bilingual language expert. A second bilingual specialist, who was not in the initial translation, independently back-translated the Arabic version into English. The original and back-translated versions were then precisely compared to detect and resolve any discrepancies in meaning, wording, and conceptual interpretation. After the completion of the review process, the final Arabic version was submitted online via Google Forms during November and December 2025. A non-probability convenience sampling strategy, supplemented by snowball dissemination, was used to recruit undergraduates. The survey link was initially distributed to academic contacts affiliated with the participating universities and subsequently circulated via university email, WhatsApp groups, and social media networks. These colleagues disseminated the invitation via university email, social media platforms, and WhatsApp groups.

Several technical and statistical measures were applied to calculate and minimize the potential impact of common method bias (CMB). Respondents were informed that their answers would remain confidential and anonymous. In addition, the measurement variables were adapted from previously validated scales and subjected to a systematic translation and back-translation procedure to improve conceptual clarity and reduce uncertainty. Harman’s single-factor test was subsequently implemented with exploratory factor analysis and an unrotated option. The first factor accounted for 36% of the overall variance, which is below the commonly applied 50% criterion (Lindell & Whitney, 2001). These results suggested that a single factor did not account for the majority of the covariance among the study variables and that common method bias was unlikely to represent a substantial threat to the study results.

## 4. Results

As advised by Henseler et al. (2009), a two-step PLS-SEM analysis of the obtained data was adopted. The first step was conducted to test the measurement model’s validity and reliability (outer model) before the second step, which tested the hypotheses (inner model).

### 4.1. Phase # One: Measurement Model Validity and Reliability

As recommended by several authors (Chin et al., 2003; Hair et al., 2021; Leguina, 2015), the scale convergent validity (the level to which different variables measuring the same factor share a high proportion of common variance) is established when the factor loadings are greater than 0.700, and AVE greater than 0.50, as shown in Table 1 all scale item factor loadings are ranged from 0.747 to 0.986 exceeding the 0.70 benchmark as suggested by (Hair et al., 2021). The AVE values ranged from 0.662 to 0.943, exceeding the threshold of 0.5 and signifying good convergent validity.

Scale reliability is established when the composite reliability (CR) exceeds 0.7 (Leguina, 2015). As depicted in Table 1, the CR values ranged from 0.899 to 0.980, indicating good scale reliability. Furthermore, two metrics were employed to measure the scale discriminant validity (the level to which a latent factor is empirically distinct from other factors in the study model) (Chin, 1998): first, the square root of the AVE values should be greater than any of the inter-factor correlations; as shown in Table 2, the AVE squared root values (diagonal bold values) are greeted that its inter factor correlation, supporting adequate discriminant validity. Second, the value of the HTMT (HTMT criteria compare the correlations between items of different factors with those among items of the same factor), as shown in Table 3, is below 0.85, further supporting good discriminant validity. Additionally, to further evaluate potential multicollinearity in the study model, the VIF values were inspected. All VIF values, as shown in Table 1, were below the recommended threshold of <3.0 (Chin, 1998), indicating that multicollinearity among the predictors was not a concern and that the estimated structural relationships were not substantially affected by collinearity.

### 4.2. Phase # Two: Inner Model for Hypothesis Testing

**Figure 1:**
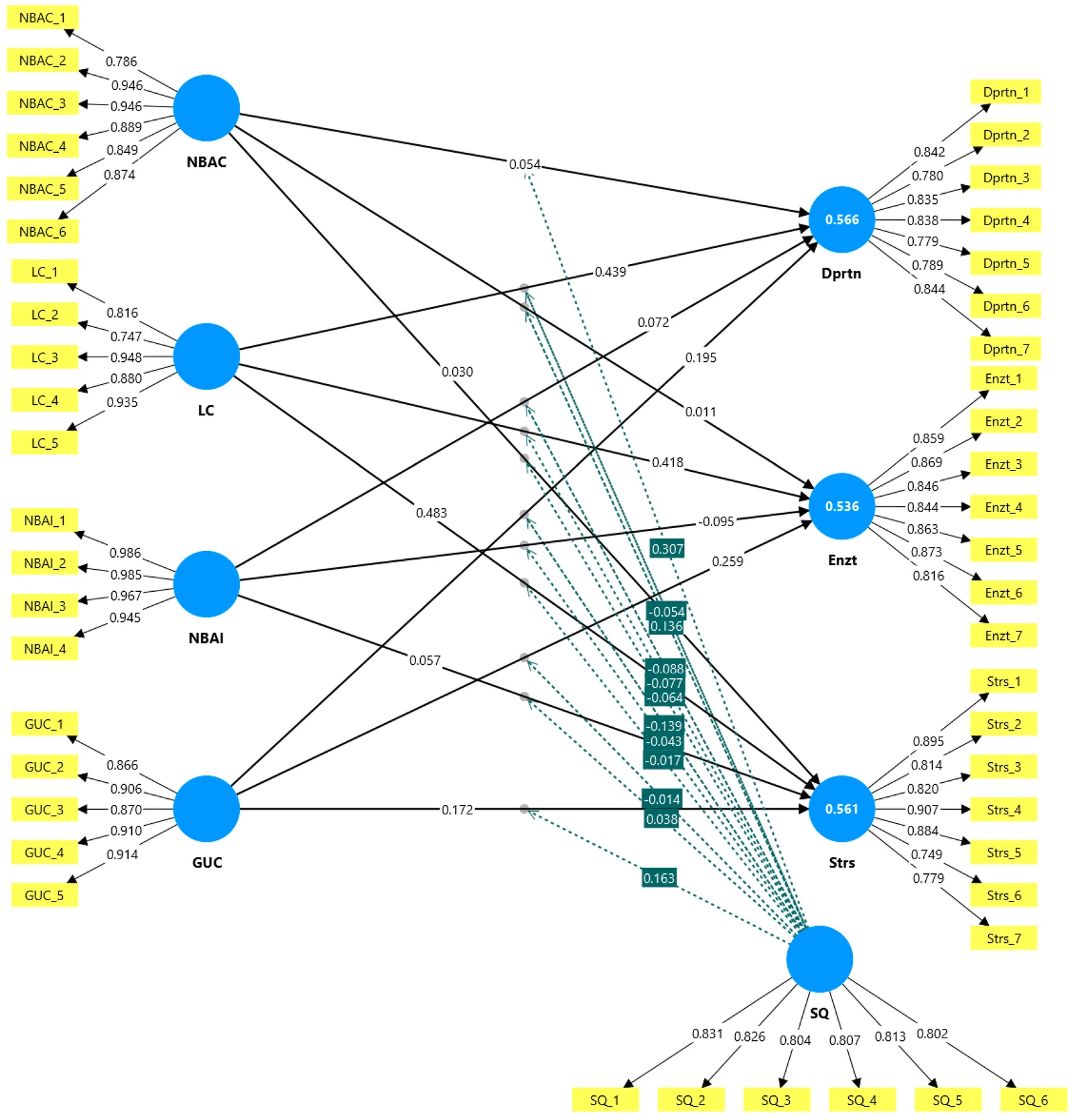
Evaluation of the structure model.

Before evaluating the study hypotheses in the inner model, the entire structural model should be assessed using predictor collinearity (VIF), explained variance (R2), effect sizes (f2), and predictive relevance (Q2) (Hair et al., 2021). Collinearity was assessed using the variance inflation factor (VIF), with scores below the adopted threshold (<3.0) indicating the absence of problematic multicollinearity among the predictors (Hair et al., 2021). Regarding R^2^ criteria (explaining how much of the endogenous variable’s variance can be explained by the exogenous variables). The model results, as shown in Figure 1, displayed R2 scores of 0.566 for depression, 0.536 for anxiety, and 0.642 for stress, all exceeding 0.10, signifying a good predictive ability of the developed model (Falk & Miller, 1992). The Q^2^ values of predictive relevance were also inspected. As suggested by Hair et al. (2021), a Q2 value above 0.0 indicates good predictive relevance. The Q^2^ values for depression, anxiety, and stress were 0.554, 0.525, and 0.526, respectively, indicating that the study model has meaningful predictive relevance. The effect size (f^2^) also ranged from 0.102 to 0.132 for the significant relationships as shown in Table 4, which is above the 0.02 threshold for a small effect (Cohen, 2013). Finally, we then tested the significance of the path coefficients using a bootstrap analysis with 5000 resamples as shown in Table 4.

The previously hypothesized relationships were evaluated using PLS-SEM and the SmartPLS program v4 to estimate the values of path coefficients (β), t-values (*t*), and p-values (*p*). The findings are shown in Table 4. The statistical significance was assessed at the 5% level.

Not being able to communicate (NBAC) as a dimension of nomophobia did not significantly contribute to increasing any of the three MH symptoms among university students. More specifically, the association between NBAC and depression (β=0.054, t=0.915, p=0.360), anxiety (β=0.011, t=0.188, p=0.851), and stress (β=0.030, t=0.474, p=0.635) were found to be insignificant. Therefore, H1, H2, and H3 were rejected. However, losing connectedness (LC), a dimension of nomophobia, showed a significant positive correlation with all three MH symptoms among university students. Specifically, LC significantly contributed to increasing the symptoms of depression (β=0.439, t=9.897, p<0.001), anxiety (β=0.418, t=9.253, p<0.001), and stress (β=0.483, t=12.060, p<0.001). Therefore, H4, H5, and H6 were confirmed. The contributions of not being able to access information (NBAI) as a dimension of nomophobia varied across the MH symptoms. NBAI was found to have a positive and significant correlation with depression (β=0.072, t=1.961, p=0.050), which marginally supported the direct association between NBAI and depression. The 95% bias-corrected and accelerated confidence interval barely encompassed zero [−0.007, 0.140], and the effect size was (f^2^ = 0.102). While this relationship is on the threshold of statistical significance, it suggests a potential trend warranting further investigation. However, NBAI showed an adverse and significant association with anxiety (β=−0.095, t=2.561, p=0.010). As depicted in Table 4, NBAI had a significant but negative association with anxiety (β = −0.095, *t* = 2.561, *p* = 0.010), with a bootstrap confidence interval ranging from −0.167 to −0.021. However, the effect size was very small (f^2^ = 0.006), implying that although the association was statistically significant, its practical magnitude was limited. Importantly, inspecting the bivariate association between NBAI and anxiety showed a positive association (r = 0.506). In contrast, the structural path coefficient became negative after the other factors, nomophobia and sleep quality, were concurrently incorporated into the model. This difference is important because the estimated path coefficient signifies the unique association of NBAI with anxiety after accounting for the shared variance with the other dimensions, rather than a simple bivariate correlation. The absence of problematic multicollinearity in the study model and the adequate discriminant validity between NBAI and anxiety support the model’s statistical estimation. In contrast, NBAI association with stress was not significant (β=0.057, t=1.557, p=0.120). Thus, H7 was marginally supported while H8 and H9 were not.

Finally, giving up convenience (GUC) as a dimension of nomophobia was positively correlated with all three MHS outcomes. Significant associations were detected for depression symptoms (β=0.195, t=3.666, p<0.001), anxiety symptoms (β=0.259, t=4.407, p<0.001), and stress symptoms (β=0.172, t=2.733, p=0.006). Therefore, H10, H11, and H12 were supported.

**Figure 2:**
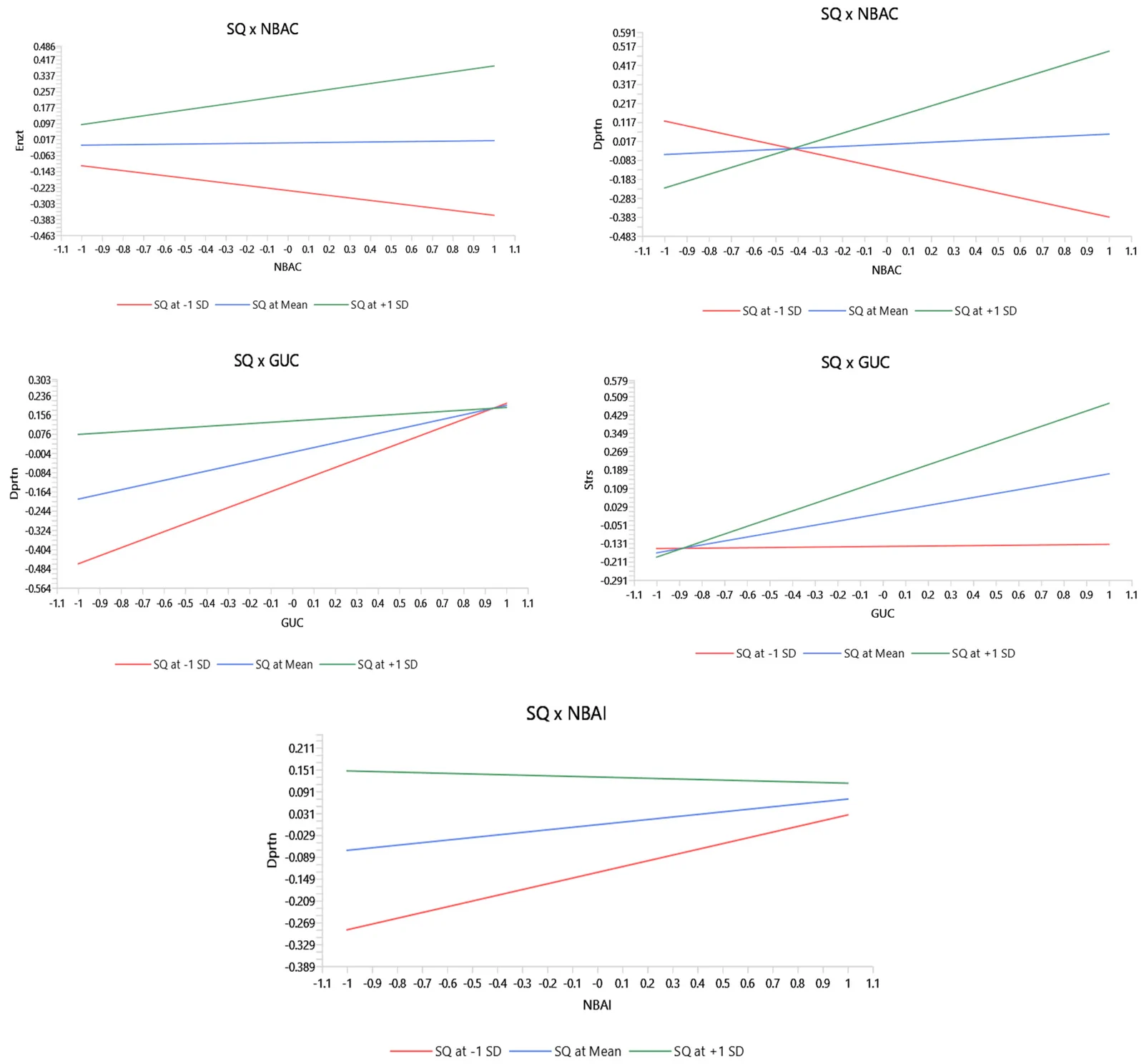
Moderation effects of SQ.

Regarding the moderation effects, the analysis provides evidence that sleep quality (SQ) significantly moderates five specific associations between dimensions of nomophobia and mental health symptoms. Specifically, the moderating effects of sleep quality (SQ), as shown in Figure 2, were examined using interaction terms between SQ and each exogenous variable. The findings indicate that sleep quality successfully and significantly can moderate two of the relationships involving NBAC. More specifically, the interaction between SQ and NBAC was found to be significant for depression (β=0.307, t=4.427, p<0.001) and anxiety (β=0.136, t=2.202, p=0.028). But the SQ × NBAC interaction was insignificant for stress (β=−0.064, t=0.812, p=0.417). Hence, H13a and H13b were supported; however, H13c was not. None of the interactions between SQ and LC was significant. Therefore, H13d, H13e, and H13f were not supported. The interaction between SQ and NBAI was significant only for depression (β=−0.088, t=1.970, p=0.049). While the other interactions were not supported. Thus, H13g was supported, whereas H13h and H13i were rejected. Regarding GUC, SQ significantly moderated its correlation with depression (β=−0.139, t=2.222, p=0.026) and stress (β=0.163, t=2.113, p=0.035). However, the interaction effect for anxiety was insignificant (β=−0.014, t=0.232, p=0.816). Accordingly, H13j and H13l were supported, whereas H13k was not supported.

## 5. Discussion and Implications

This study tested the association between four nomophobia dimensions (NBAC, LC, NBAI, and GUC) and three mental health symptoms (Dprtn, Enzt, and Strs), while investigating sleep quality (SQ) as a key moderator. The strongest and most consistent finding was the positive association between losing connectedness and all the three dimensions of MHS (depression, anxiety, and stress). This pattern suggested that losing connectedness might be particularly significant for university students, as the higher education environment may involve substantial social and developmental challenges (Elshaer et al., 2026b). Students might be separated from their families, adjust to new academic settings, form new peer connections, and rely on social relationships for academic and emotional support (Kumar et al., 2025). The current results are consistent with a previous longitudinal study conducted by Li et al. (2020), which reported that social isolation and loneliness among college students can predict subsequent depressive symptoms. It is also consistent with previous evidence reporting that social disconnectedness might contribute to higher levels of perceived isolation, which successively can predict depression and anxiety (Santini et al., 2020). The positive association between LC and the three dimensions of MHS can also be understood through the role of belonging in students’ university life. University students who feel disconnected from classmates, peers, lecturers, or other family members might have fewer opportunities to gain support, exchange academic responsibilities, manage emotions, and receive practical support (Rawas et al., 2021). Consequently, LC might function as a primary psychosocial stressor that can simultaneously contribute to Dprtn, Enzt, and Strs. Giving up convenience (GUC) was found to be significantly and positively associated with increased depression, anxiety, and stress symptoms. These results suggest that convenience loss might be emotionally relevant to university students when it interferes with academic pressures, interactions, mobility, access to university services, or everyday challenges (Naser et al., 2023). University students regularly try to balance academic challenges with family and social responsibilities (Demirci et al., 2015). Under these settings, losing a convenient service or a well-established routine might increase perceived effort and reduce students’ sense of control. The relationship might be specifically evident during exam times, assignment closing dates, or transition periods.

Not being able to communicate (NBAC) was found to be insignificantly associated with mental health symptoms (depression, anxiety, and stress). These results indicate that communication problems, when evaluated as a direct predictor, might be insufficient in predicting the university student’s psychological distress. Students might compensate through alternative channels, such as face-to-face communication, learning management systems, or institutional support services. Similarly, the association between not being able to access information (NBAI) was found to vary across the three MH symptoms. NBAI was found to have a small positive correlation with depression symptoms among university students but failed to predict stress significantly. However, NBAI demonstrated a significant but negative association with anxiety. This unexpected association might have several interpretations in a university-student setting. Access to information might sometimes increase the level of anxiety when university students are subjected to extreme, ambiguous, terrifying, or inconsistent information (Rodríguez-García et al., 2020). One more possible justification for this counterintuitive association is that smartphone-based information access might have a dual emotional function. Although failure to access information might create uncertainty and discomfort, constant access to online information might also expose university students to excessive, contradictory, or uncertainty-inducing information. Therefore, once the overlapping correlations between losing connectedness, communication difficulties, giving up convenience, and sleep quality are statistically controlled, the information-specific dimension of nomophobia might exhibit a small negative unique correlation with anxiety. However, this justification remains tentative because the cross-sectional design did not permit causal inference. On the contrary, lower level of exposure to stressful information might be correlated with lower level of anxiety for some university students. The relationship might also be understood in terms of whether the information is academic, health-related, or social, and whether university students perceive it as valid and reliable.

Regarding the moderation effects, the moderation analysis provides evidence that sleep quality (SQ) can significantly moderate specific associations between selected dimensions of nomophobia and MHS. Only five statistically significant interaction paths were detected: SQ moderated the association between NBAC and depression, NBAC and anxiety, NBAI and depression, GUC and depression, and GUC and stress. Significantly, the direction of these interactions’ effects was not uniform. This pattern suggested that SQ should not be theorised simply as a universal protective element; rather, the moderating role appears to depend on the specific dimension of nomophobia and MHS under consideration. The interaction slope between SQ and NBAC was positive and significant in predicting depression symptoms among university students. This was the largest moderation effect among the five significant interactions, implying that SQ can alter the correlation between NBAC and depressive symptoms. Similarly, SQ can significantly moderate the associations between NBAC and anxiety. Though the moderation was smaller than was detected for depression, it still shows that the moderating role of SQ can extend beyond depressive to anxiety symptoms.

This result indicated that the positive correlation from NBAC to depression and anxiety symptoms becomes greater as sleep quality improves. This result is theoretically challenging and unexpected because it did not support the established assumption that better SQ necessarily attenuates the emotional outcomes of smartphone dependence. Instead, it suggested that the association between NBAC and depressive and anxiety symptoms is conditional upon SQ in a more complex manner. One possible justification concerns the unique emotional content of NBAC. This nomophobia factor describes concerns that accompany being unable to communicate through a smartphone. For university students, smartphones have become deeply embedded in their daily interpersonal relationships, academic challenges, family interactions, and social involvement. Subsequently, failure to communicate might represent more than a temporary technological problem; for people with strong reliance on continuous connectivity, it might threaten perceived social convenience and interpersonal protection.

A different moderation form appeared for GUC. The interaction between GUC and SQ was negative and significant in increasing depression symptoms. This finding indicated that SQ can alter the strength of the GUC–depression association. The negative interaction slope indicates that better SQ decreases the positive correlation between GUC and depression symptoms. In other words, the emotional burden associated with losing the convenience provided by smartphones seems to be more evident among students with poorer SQ and less evident among those with better SQ. This result provides strong confirmation of the theoretical view of SQ as a psychological asset. When SQ is poor, university students might have fewer cognitive and psychological resources available to cope with such disruptions. Previous literature has confirmed a correlation between smartphone overuse, sleep problems, and emotional distress. The current findings indicate that the mental-health implications of smartphone convenience are not the same for university students. Instead, they appeared to be moderated by SQ. Good SQ might confer greater psychological and self-regulatory capacity, enabling students to adapt more successfully when smartphone functionality is unavailable. Poor SQ, by contrast, might leave university students more vulnerable to the psychological effects of disrupted routines and a sense of loss of control.

Similarly, SQ can also significantly moderate the relationship between GUC and stress. This is the second major significant interaction and demonstrates that the relationship between reliance on smartphone convenience and perceived stress varies according to SQ. Interestingly, this unexpected positive interaction indicated that the relationship between GUC and stress can be stronger when sleep quality improves. One probable interpretation is that GUC and stress might function through the perceptions of loss of control and functional disruption. University students with good SQ may have more stable daily responsibilities and may depend heavily on smartphones to manage, direct, and optimise them. Subsequently, as smartphone convenience fades, the disruption might be particularly salient and manifest as stress. On the contrary, university students already experiencing poor SQ might confront multiple competing sources of emotional and functional difficulty, theoretically making the additional inconvenience correlated with smartphone loss less effective.

Although COR Theory and the Hyperarousal Model of Insomnia predicted that poor sleep quality would strengthen the adverse psychological consequences of nomophobia, the observed interaction associations involving NBAC and GUC were in the opposite direction. Specifically, the significant SQ × NBAC interactions for depression and anxiety and the significant SQ × GUC interaction for stress suggested that higher SQ was correlated with stronger, rather than weaker, associations between these nomophobia dimensions and selected mental health symptoms. One probable explanation is that SQ might act as a context-dependent resource rather than as a universally protective buffer. University students who sleep relatively well might have greater cognitive alertness and psychological capacity to identify disruptions in communication, responsiveness, or everyday convenience, making the perceived loss of these resources more salient when they happen. By contrast, university students undergoing poor sleep might report more diffuse fatigue and psychological distress, potentially reducing the distinctive contribution of a specific nomophobia dimension. Nevertheless, this possible explanation remains tentative. The results should consequently be interpreted as exploratory, context-specific interaction effects rather than evidence that good sleep causes depression, anxiety, or stress or that poor sleep is protective.

Finally, the insignificant moderation paths indicated that SQ was not capable of significantly altering several relationships between nomophobia and MH symptoms among university students. Specifically, SQ did not moderate the relationship between NBAC and stress; LC and depression, anxiety, or stress; NBAI and anxiety or stress; or GUC and anxiety. These results suggested that, among university students, SQ did not regularly alter the strength or direction of these specific associations, even though it might have significant moderating interactions in the other parts of the model. Taken together, these results indicate that SQ is not a universal protective factor against the emotional outcomes of nomophobia. Instead, its moderating effects are dimension-specific, affecting some types of smartphone reliance while leaving other associations unchanged. These results advanced previous literature, which has mainly conceptualised SQ as either an outcome of nomophobia or a moderator of the relationship between smartphone overuse and MHS.

The study results provided several theoretical and practical implications for understanding emotional distress among university students. First, losing connectedness (LC) as a dimension of nomophobia was the most consistent predictor of depression, anxiety, and stress symptoms, signifying that social and interpersonal disturbance might be more emotionally significant than communication or information challenges considered in isolation. This result supported the social-connection views, which posit that belonging, relational contact, and perceived support are influential resources for emotional adjustment. In the university environment, LC might involve reduced contact with relatives, peers, colleagues, or university populations, thereby decreasing psychological support and aggregate exposure to distress. Previous research has linked loneliness and social isolation with severe depressive symptoms among university students (Chandler et al., 2022), while several evidence indicated that social disconnectedness might increase personal isolation and successive anxiety and depression (Santini et al., 2020). Additionally, the significant associations between GUC and MH symptoms (Dprtn, Enzt, and Strs) signaled that practical disturbance should be merged into student mental health-related models. Students at university regularly manage academic due dates, exam duties, employment, and family commitments; consequently, the loss of convenient services or routines might increase perceived challenges and reduce self-control. Furthermore, the insignificant associations between NBAC and MH symptoms signaled that communication restrictions might not be able to independently predict emotional distress. Their associations might instead function indirectly through other factors such as social support or academic disruption. Finally, SQ showed a selective moderating role: it significantly moderated some associations, such as NBAC, NBAI, and GUC, but not others, such as LC with Dprtn, Enzt, or Strs, or several other proposed slopes. The findings therefore did not confirm that SQ is a common protective factor; rather, its effects appear to depend on the specific predictor and emotional outcome. This justification is aligned with university-student-related research indicating that SQ and stress are closely correlated, while also signifying that sleep-related mechanisms might function differently across student experiences and mental-health consequences (Chandler et al., 2022).

Practically, the study’s findings indicated that higher education institutions should implement a student-centred, integrated mental-health program that aims to address social interactions, academic disruption, information control, and sleep quality. First, as LC was found to have a significant association with all three MH outcomes, universities should develop strategies that encourage belonging and social interaction, including peer-mentoring programs, first-year transition policies, student-led communities, and academic culture groups. These programs should offer not only social services but also academic collaboration, psychological support, and consistent contact with student consultants. Recent research suggested that social-interaction programs might create small but significant improvements in depression among adults (Lin et al., 2021). Second, the positive associations between GUC and MH symptoms indicated that universities should reduce unnecessary administrative and service-related inconveniences. Institutions can successfully implement this by preserving alternative two-way communication channels, providing clear instructions during system failures, providing timely technical support, shortening and simplifying student service processes, and permitting reasonable flexibility when university disruptions affect academic duties. Third, university student-support services should incorporate SQ evaluation and evidence-based sleep interventions, especially during examination periods and major academic challenges. This suggestion is supported by evidence that sleep interventions among university students can reduce sleep interruptions and yield small improvements in depression and anxiety symptoms (Chandler et al., 2022), while emotional interventions can improve sleep quality among university students (Tadros et al., 2025). However, as several SQ moderation slopes were insignificant, university leaders should not propose that enhancing SQ levels will reduce the impacts on social withdrawal, communication restrictions, or information-access issues. Instead, sleep interventions should supplement, rather than substitute, social and academic assistance and institutional-reliability procedures.

## 6. Limitations and Future Study Opportunities

This study has several limitations, like those of previous studies in the social sciences, which should be acknowledged when explaining its results. These limitations can offer opportunities for future related research. First, the study is cross-sectional, which may limit the ability to infer causal associations between the study variables. Future research should adopt longitudinal research designs to establish causal relationships. Second, all employed scales were measured using self-reported surveys, making the results sensitive to common method variance (CMV). Although several statistical and procedural remedies were implemented to minimise CMV, future research can employ a multi-method approach to improve measurement validity. Third, the study was conducted entirely with university students; consequently, the generalizability of the results to other segments or populations remains uncertain. Future research should replicate the developed model across diverse groups to determine whether the associations identified in this research are stable across contexts. Fourth, the study tested sleep quality as the only moderating variable. Future research should investigate additional boundary factors, such as self-efficacy, emotional resilience, social support, self-control, and emotional intelligence, that may strengthen or weaken the associations between nomophobia and MHS. The sampling strategy represented an important limitation. Participants were recruited through nonprobability convenience sampling and snowball sampling via university networks, WhatsApp groups, social media platforms, and academic contacts. Consequently, students who are more digitally active, more socially connected, or more willing to participate in online research may be overrepresented. Future research should use probability-based, stratified, or institutionally sampled designs, record survey exposure and completion metrics, recruit students through multiple channels beyond digital social networks, and compare respondents with nonrespondents where possible. The demographic composition of the study sample can be considered another concern. Although applicants were from five SA universities representing different geographical regions and academic disciplines, the sample was primarily composed of younger university students, with 90% aged 17–21 years. In addition, males represented 60% of the sample, compared with 40% females. This distribution might limit the generalizability of the findings. Future research can employ stratified or probability-based sampling to obtain more balanced representation across age groups and genders.

## Figures and Tables

**Table 1:** Validity and reliability results.

Variables Items	Factor Loadings	Alpha	CR	AVE	VIF
Depression (Dprtn)		0.916	0.918	0.665	
Dprtn_1	0.842				3.15
Dprtn_2	0.780				2.03
Dprtn_3	0.835				3.17
Dprtn_4	0.838				2.26
Dprtn_5	0.779				2.46
Dprtn_6	0.789				2.36
Dprtn_7	0.844				2.76
Anxiety (Enzt)		0.938	0.940	0.728	
Enzt_1	0.859				2.50
Enzt_2	0.869				2.51
Enzt_3	0.846				2.12
Enzt_4	0.844				1.25
Enzt_5	0.863				2.91
Enzt_6	0.873				2.47
Enzt_7	0.816				2.172
Giving up convenience (GUC)		0.937	0.939	0.798	
GUC_1	0.866				2.76
GUC_2	0.906				2.54
GUC_3	0.870				2.97
GUC_4	0.910				2.35
GUC_5	0.914				1.91
Losing connectedness (LC)		0.917	0.929	0.754	
LC_1	0.816				2.17
LC_2	0.747				1.70
LC_3	0.948				1.08
LC_4	0.880				2.83
LC_5	0.935				1.47
Not being able to communicate (NBAC)		0.943	0.947	0.781	
NBAC_1	0.786				1.88
NBAC_2	0.946				2.92
NBAC_3	0.946				1.28
NBAC_4	0.889				1.76
NBAC_5	0.849				3.01
NBAC_6	0.874				1.47
Not being able to access information (NBAI)		0.980	0.982	0.943	
NBAI_1	0.986				1.31
NBAI_2	0.985				2.06
NBAI_3	0.967				1.51
NBAI_4	0.945				2.03
Sleep quality (SQ)		0.898	0.899	0.662	
SQ_1	0.831				2.24
SQ_2	0.826				2.90
SQ_3	0.804				2.20
SQ_4	0.807				1.42
SQ_5	0.813				2.96
SQ_6	0.802				3.02
Stress (Strs)		0.928	0.929	0.701	
Strs_1	0.895				1.90
Strs_2	0.814				1.78
Strs_3	0.820				1.93
Strs_4	0.907				1.01
Strs_5	0.884				1.581
Strs_6	0.749				1.51
Strs_7	0.779				1.33

Notes: AVE: “Average Variance Extracted”; CR: “Composite Reliability”.

**Table 2:** Discriminant validity-Fornell–Larcker Criterion.

	Dprtn	Enzt	GUC	LC	NBAC	NBAI	SQ	Strs
Dprtn	**0.816**							
Enzt	0.707	**0.853**						
GUC	0.549	0.563	**0.894**					
LC	0.694	0.654	0.513	**0.869**				
NBAC	0.556	0.560	0.290	0.556	**0.884**			
NBAI	0.593	0.506	0.502	0.765	0.529	**0.971**		
SQ	0.620	0.644	0.581	0.740	0.588	0.618	**0.814**	
Strs	0.643	0.617	0.530	0.707	0.538	0.604	0.632	**0.837**

Notes: Bold values in the diagonal represent the √ AVE values; GP, Depression (Dprtn); Anxiety (Enzt); Giving up convenience (GUC); Losing connectedness (LC; Not being able to communicate (NBAC); Not being able to access information (NBAI); Sleep quality (SQ); Stress (Strs).

**Table 3:** HTMT ratio for discriminant validity results.

	Dprtn	Enzt	GUC	LC	NBAC	NBAI	SQ	Strs
Dprtn								
Enzt	0.759							
GUC	0.589	0.594						
LC	0.752	0.699	0.549					
NBAC	0.594	0.587	0.773	0.595				
NBAI	0.625	0.527	0.520	0.700	0.549			
SQ	0.680	0.696	0.632	0.719	0.637	0.657		
Strs	0.700	0.657	0.565	0.765	0.570	0.632	0.689	

Notes: Depression (Dprtn); Anxiety (Enzt); Giving up convenience (GUC); Losing connectedness (LC); Not being able to communicate (NBAC); Not being able to access information (NBAI); Sleep quality (SQ); Stress (Strs).

**Table 4:** Results of hypothesis testing.

Hypotheses	β	T-Values	*p* Values	f^2^	Bootstrap Confidence Intervals		Results
					2.5%	97.5%	
**H1. NBAC -> Dprtn**	0.054	0.915	0.360	0.001	−0.068	0.164	Not confirmed
**H2. NBAC -> Enzt**	0.011	0.188	0.851	0.000	−0.111	0.126	Not confirmed
**H3. NBAC -> Strs**	0.030	0.474	0.635	0.012	−0.097	0.149	Not confirmed
**H4. LC -> Dprtn**	0.439	9.897	0.000	0.107	0.349	0.524	Confirmed
**H5. LC -> Enzt**	0.418	9.253	0.000	0.132	0.327	0.502	Confirmed
**H6. LC -> Strs**	0.483	12.060	0.000	0.133	0.405	0.562	Confirmed
**H7. NBAI -> Dprtn**	0.072	1.961	0.050	0.102	0.002	0.149	Confirmed
**H8. NBAI -> Enzt**	−0.095	2.561	0.010	0.006	−0.167	−0.021	Not confirmed
**H9. NBAI -> Strs**	0.057	1.557	0.120	0.005	−0.014	0.131	Not confirmed
**H10. GUC -> Dprtn**	0.195	3.666	0.000	0.113	0.094	0.304	Confirmed
**H11. GUC -> Enzt**	0.259	4.407	0.000	0.121	0.150	0.382	Confirmed
**H12. GUC -> Strs**	0.172	2.733	0.006	0.102	0.050	0.295	Confirmed
**Moderation**							
**H13a. SQ × NBAC -> Dprtn**	0.307	4.427	0.000	0.122	0.170	0.443	Confirmed
**H13b. SQ × NBAC -> Enzt**	0.136	2.202	0.028	0.104	0.012	0.254	Confirmed
**H13c. SQ × NBAC -> Strs**	−0.064	0.812	0.417	0.004	−0.214	0.098	Not confirmed
**H13d. SQ × LC -> Dprtn**	−0.054	0.937	0.349	0.002	−0.160	0.064	Not confirmed
**H13e. SQ × LC -> Enzt**	−0.077	1.716	0.086	0.003	−0.166	0.011	Not confirmed
**H13f. SQ × LC -> Strs**	−0.017	0.367	0.714	0.000	−0.105	0.073	Not confirmed
**H13g. SQ × NBAI -> Dprtn**	−0.088	1.970	0.049	0.104	−0.174	−0.004	Confirmed
**H13h. SQ × NBAI -> Enzt**	−0.043	0.990	0.322	0.001	−0.132	0.039	Not confirmed
**H13i. SQ × NBAI -> Strs**	0.038	0.803	0.422	0.000	−0.057	0.128	Not confirmed
**H13j. SQ × GUC -> Dprtn**	−0.139	2.222	0.026	0.140	−0.267	−0.016	Confirmed
**H13k. SQ × GUC -> Enzt**	−0.014	0.232	0.816	0.005	−0.132	0.109	Not confirmed
**H13l. SQ × GUC -> Strs**	0.163	2.113	0.035	0.102	0.011	0.315	Confirmed

Notes: Depression (Dprtn); Anxiety (Enzt); Giving up convenience (GUC); Losing connectedness (LC); Not being able to communicate (NBAC); Not being able to access information (NBAI); Sleep quality (SQ); Stress (Strs). Sleep quality (SQ).

## Data Availability

The original contributions presented in the study are included in the article; further inquiries can be directed to the corresponding author.
